# Influence of aluminum and iron chlorides on the parameters of zigzag patterns on films dried from BSA solutions

**DOI:** 10.1038/s41598-023-36515-4

**Published:** 2023-06-09

**Authors:** Dmitriy Glibitskiy, Olga Gorobchenko, Oleg Nikolov, Tatyana Cheipesh, Tatyana Dzhimieva, Inna Zaitseva, Alexander Roshal, Mihail Semenov, Gennadiy Glibitskiy

**Affiliations:** 1 O. Ya. Usikov Institute for Radiophysics and Electronics, National Academy of Sciences of Ukraine, 12 Academician Proskura Str., Kharkiv, 61085 Ukraine; 2 V. N. Karazin Kharkiv National University, 4 Svobody Sq., Kharkiv, 61022 Ukraine; 3 O. M. Beketov National University of Urban Economy in Kharkiv, 17 Marshal Bazhanov Str., Kharkiv, 61002 Ukraine; 4 Institute for Chemistry, V. N. Karazin Kharkiv National University, 4 Svobody Sq., Kharkiv, 61022 Ukraine

**Keywords:** Biophysical methods, Biological fluorescence

## Abstract

The relationships between the structural and aggregational state of bovine serum albumin (BSA) and the specific length and total number of zigzag pattern segments of the film textures formed upon drying biopolymer solutions with aluminum and iron chlorides have been shown. To obtain films, saline solutions of BSA were dried in a glass cuvette under thermostatically controlled conditions. It is shown that the formation of zigzag structures is sensitive to the influence of aluminum chlorides Al^3+^ and iron chlorides Fe^3+^ and depend on the concentration of AlCl_3_ and FeCl_3_. This may be due to a change in the charge and size of BSA particles and due to a change in conformation or a violation of the structure of BSA. These factors, in turn, affect the hydration of the solution components and the structural state of free water in solution, which presumably also affects the formation of zigzag structures. It is established that the analysis of the specific length and the number of segments of zigzag patterns makes it possible to evaluate changes in the state of biopolymers in the initial solution during structural changes and aggregation.

## Introduction

Changes in textures on biopolymer films reflect the interaction with metal ions in the initial solution, and, as a simplified model system, can give an idea of the nature of the interaction in vivo. AlCl_3_ is a well-known coagulant and is used, in particular, for wastewater treatment from organic matter and colloidal particles^1^. The hydrolysis products of aluminum and iron salts change pH and can be deposited on negatively charged surfaces and surround colloidal particles; positively charged ions can lead to coagulation of colloidal particles due to the compensation of the surface potential^2,3^. It has been shown that electrostatic interaction is the determining factor in the adsorption of model proteins, except for ovalbumin^4^.

The interaction of aluminum and iron ions with bovine serum albumin (BSA) has been studied by various spectroscopic methods in recent years. UV–Vis absorption spectra along with infrared spectra were used to measure Al^3+^-BSA binding constants^5^. UV, Raman and circular dichroism spectroscopy of the complexes makes it possible to draw conclusions about the nature of the interaction of Fe^3+^ with BSA, namely, with tryptophan and tyrosine residues^6^.

Studies of the molecular mechanisms of interaction with iron ions^7^ have shown that BSA has a specific Fe^3+^-binding site adjacent to both tryptophan residues in BSA. It was concluded that Fe^2+^ and Fe^3+^ ions have no obvious effects on the secondary structure of BSA and Fe^3+^ ions bind to tryptophan residues in BSA. The study of the interaction of BSA with aluminum ions has shown that, depending on the concentration of aluminum, up to three Al^3+^ ions^8^ bind to BSA at two coordination sites^9^. The work^2^ noted the interaction of Al^3+^ and Fe^3+^ with charged side chains of amino acids on the protein surface. According to^10^, aluminum ions form backbone ring structures leading to protein denaturation.

It was shown in^2^ that the binding of multivalent ions and changes in the pH of the protein solution due to the hydrolysis of metal salts play an important role in the surface charge of the protein. Studies of the dependence of the properties of BSA in solution on the pH value have shown that the isomeric form of albumin at pH values below 4.0 ("F" form) corresponds to the sequential subdomain-subdomain dissociation, accompanied by the loss of intradomain helices, and at pH less than 3.5, the macromolecule acquires an extended configuration ("E" form)^11,12^. Lowering the pH of the solution leads^13^ to partial denaturation of albumin, exposing the hydrophobic regions of the core, and to aggregation with the formation of connected structures, and may be the basis for self-assembly of the hydrogel percolation network^14^. Long-range interactions in BSA molecules cease to be observed when pH approaches the isoelectric point (pI) of BSA^15^. Studies have also shown that protein adsorption on the surface of the cuvette substrate strongly depends on pH^16^, and that one of the BSA aggregation factors is the intermolecular exchange of disulfide through the cysteine residue^17^.

When droplets of biological fluids and solutions of biopolymers are dried, a film is formed on the surface of the substrate, the texture of which has parameters that reflect the properties of solutes and the nature of their interaction. The data obtained from the analysis of the textures of such films can be used in medical diagnostics^18–20^ and in molecular biology for screening biologically active substances (BAS)^21^. In such studies, much attention is also paid to the analysis of fundamental processes associated with the drying of the droplet and to the determination of the mechanisms of pattern formation^22–24^.

The nature of the textures is determined by the dynamic self-assembly mechanism, fluid flows and the particle transfer in the drying drop^25–28^. Various authors apply methods for analyzing patterns and modeling their formation, based on the parameters of the statistics of intensity values^29^, diffusion-limited aggregation^30^, molecular dynamics^31^, Monte Carlo^32,33^ and using machine learning^34^. Based on the Z-patterns (zigzag patterns) described in^35^, we have developed a method for assessing the change in the parameters of the patterns^36^ due to the influence of chemical and physical factors on the biopolymer in the initial solution.

The purpose of this work is to study marker changes in such parameters as the specific length and total number of zigzag pattern segments on the film textures formed upon drying BSA solutions with aluminum and iron chlorides, in the direction of analysis of relationships between the structural and aggregational state of the biopolymer and these parameters.

## Results and discussion

### Film textures and their characteristics

**Figure 1:**
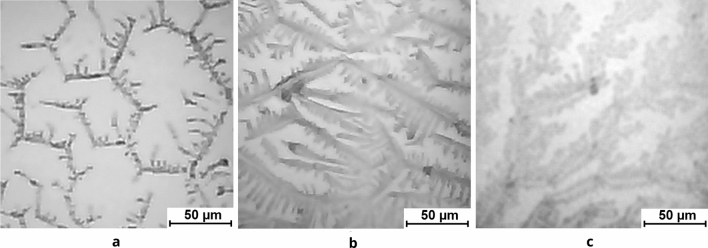
(**a**) Micrograph of the film texture obtained from a solution of 0.5 mg/ml BSA + 20 mM NaCl, (**b**) micrograph of the film texture obtained from a solution of 0.5 mg/ml BSA + 20 mM NaCl + 0.2 mM AlCl_3_, (**c**) micrograph of the film texture obtained from a solution of 0.5 mg/ml BSA + 20 mM NaCl + 0.2 mM FeCl_3_. The micrographs are fully in focus.

Example textures of films of BSA solutions with NaCl and the addition of 0.2 mM AlCl_3_ or FeCl_3_ are shown in Fig. 1. It can be seen that the presence of zigzag patterns is the most prominent on the control films (Fig. 1a) and is reduced less on the AlCl_3_ film (Fig. 1b) than on the FeCl_3_ film (Fig. 1c) at the same salt concentrations.

**Figure 2:**
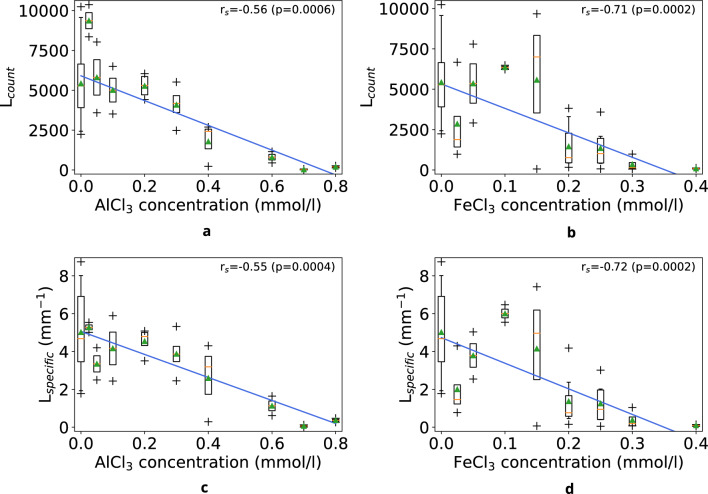
(**a**) Number of zigzag segments on the surface of films from BSA + NaCl + AlCl_3_ solutions, (**b**) number of zigzag segments on the surface of films from BSA + NaCl + FeCl_3_ solutions, (**c**) specific length of zigzag segments on the surface of films from BSA + NaCl + AlCl_3_ solutions, (**d**) specific length of zigzag segments on the surface of films from BSA + NaCl + FeCl_3_ solutions. At the top of the plots, the Spearman's rank correlation coefficient (r_s_) and its statistical significance value (p) are provided.

Figure 2 presents the dependences of the number of zigzag segments and their specific length on AlCl_3_ and FeCl_3_ concentrations in the initial solutions. As can be seen from Fig. 2a, b, the number of Z-structures decreases with increasing concentration of AlCl_3_ and FeCl_3_.

The number of segments of zigzag patterns up to 0.2 mM AlCl_3_ is within the control values and decreases starting from 0.4 mM AlCl_3_ (Fig. 2a).

It can be seen that the average L_count_ for AlCl_3_ and FeCl_3_ films at concentrations of 0.2 mM or more differ by more than three times, as can be seen from the plots showing L_count_ versus concentrations in Fig. 2a, b. The values of L_count_ decrease almost to zero at concentrations of 0.7 and 0.3 for AlCl_3_ and FeCl_3_, respectively.

Figure 2c, d show the specific lengths for AlCl_3_ and FeCl_3_ films. Like for the L_count_, these values also differ by almost three times after the concentration values of 0.7 and 0.3 for AlCl_3_ and FeCl_3_, respectively. Thus, both parameters, as criteria for the characteristics of zigzag patterns, change with increasing concentrations of AlCl_3_ and FeCl_3_, which is statistically supported by the significance p-values of the correlation (Fig. 2).

The change in the number of zigzag segments on the surface of films from BSA + NaCl + FeCl_3_ solutions (Fig. 2b) depending on the concentration shows that L_count_ has several different areas: up to 0.15 mM FeCl_3_, L_count_ is in the range of control values and even tends to grow; between 0.2 and 0.25 mM FeCl_3_, the number of zigzags noticeably decreases and fluctuates approximately in the same range; at 0.3 mM, L_count_ decreases again, and at 0.4 mM FeCl_3_, zigzags cease to form. At the same time, an increase in the concentration of FeCl_3_ in BSA solutions leads to the predominance of other types of patterns.

### Dielectric permittivity measurements

**Figure 3:**
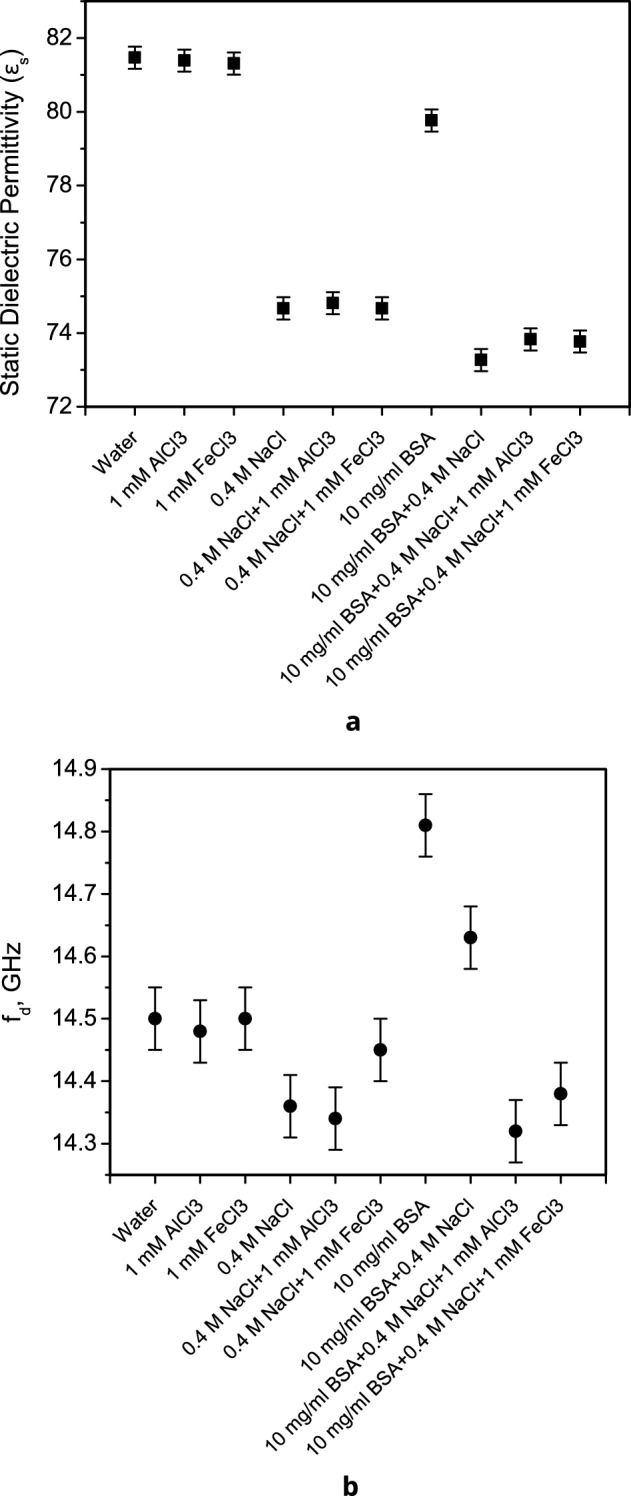
(**a**) Static permittivity ${\varepsilon }_{s}$ of aqueous solutions of salts and BSA (15.5 ± 0.1 °C); (**b**) frequency of dielectric relaxation of water molecules f_d_ in aqueous solutions of salts and BSA (15.5 ± 0.1 °C).

The state of the water molecules surrounding the BSA was investigated using microwave dielectrometry^37^. In solutions of proteins or salts, the static permittivity ${\varepsilon }_{s}$ (Fig. 3a) is determined mostly by the amount of free water. Thus, the presence of 0.4 M NaCl leads to a decrease in the value of ${\varepsilon }_{s}$ by approximately 7 units, while 10 mg/ml BSA reduces it by 1.7 units compared to pure water, and by 1.4 units compared to 0.4 M NaCl. The addition of 1 mM AlCl_3_ and FeCl_3_ to a 0.4 M NaCl solution causes BSA to decrease ${\varepsilon }_{s}$ by smaller values, 1.0 and 0.9 units, respectively. This indicates that, in the presence of these salts, the hydration of BSA in solution decreases.

The dielectric relaxation frequency f_d_ (Fig. 3b) characterizes the mobility of water molecules in solution, which, in turn, is determined by the nature of intermolecular interactions. An increase in the frequency of dielectric relaxation of water molecules f_d_ of a 10 mg/ml BSA solution in pure water indicates a decrease in the life-time of the hydrogen bonds. Hydrogen network fluctuations and the reorientation motion is initiated by the presence of an additional neighbor molecule in a position that firstly flattens the potential energy barrier for the reorientation of a given dipole moment and that simultaneously offers a site for the formation of a new hydrogen bond^38^.

One can note a tendency to a decrease in f_d_ due to the presence of 0.4 M NaCl in the solution, including in the BSA solution. In the presence of NaCl + AlCl_3_ and NaCl + FeCl_3_ salts, the dielectric relaxation frequencies of BSA solutions further decrease and take values close to the f_d_ values of the corresponding solutions of these salts. This indicates a greater structuring of free water in these samples (a reduction in their mobility) and is consistent with the increase in viscosity of the BSA solution in the presence of Fe^3+^^6^.

### pH of BSA solutions with AlCl_3_ and FeCl_3_

The pH measurement results have shown that for the BSA + NaCl control solution, pH = 6.8 ± 0.4. With an increase in the concentration of Al^3+^ and Fe^3+^ to 0.4 mM, the pH value decreases to pH = 4.4 ± 1.0 ﻿(Al^3+^) and pH = 3.1 ± 0.7 (Fe^3+^)﻿, respectively. This is due to the hydrolysis reaction of these salts (the difference in pH values for the same concentrations of aluminum and iron is due to the difference in dissociation constants of their hydroxides^2^). Calculation of particle concentration based on the equilibrium constants of hydrolysis and dissociation of hydroxy complexes of aluminium and iron ions^39,40^ has shown that in dilute solutions, most of the ferric chloride is hydrolyzed to insoluble hydroxide, which is probably distributed in the solution in the form of colloidal particles.

To investigate the effect of pH changes on the properties of BSA solutions in the range of 3.8 > pH > 2.4, BSA solutions were studied in the presence of NaCl with the addition of HCl at appropriate concentrations. Analysis of ultraviolet (UV) spectra has shown that the spectra of BSA control solutions and BSA solutions with different concentrations of HCl in the presence of NaCl practically coincide.

It can be noted that in the pH range from 3.6 to 3.4, the decrease in L_count_ and L_specific_ (AlCl_3_) is from 5000 to 1800 and from 4.2 to 2.6, respectively. In the pH range from 2.8 to 2.5, the decrease in L_count_ and L_specific_ (FeCl_3_) is from 6400 to 0 and from 6 to 0, respectively.

Thus, the change in the parameters of the spectra in the presence of iron and aluminum ions and, consequently, the nature of the effect of these ions on BSA molecules in solution, is mainly determined by the interaction of these ions with BSA, and not by changes in the pH of solutions.

### Zeta potential (${\varvec{\upzeta}}$) and BSA particle sizes according to dynamic light scattering

Table 1 presents the values of the zeta potential and the diameters of BSA particles in BSA + NaCl + AlCl_3_ and BSA + NaCl + FeCl_3_ solutions, measured by the dynamic light scattering (DLS) method, taking into account the hydration shell (hydrodynamic diameters), corresponding to the maxima of their scattering intensity (the corresponding size distributions by volume and intensity are provided in the Supplementary Fig. S1).

**Table 1:** Zeta potential and hydrodynamic diameters of BSA particles in solutions with Fe^3+^ and Al^3+^.

Solutions	Zeta potential, mV	Hydrodynamic diameter, nm
BSA + 20 mM NaCl (control)	−16.1 ± 1.4	9.4 ± 1.2*
BSA + 20 mM NaCl + 0.025 mM FeCl_3_	−14.0 ± 2.0	10.2 ± 0.4
BSA + 20 mM NaCl + 0.05 mM FeCl_3_	−12.9 ± 1.2	9.6 ± 0.8
BSA + 20 mM NaCl + 0.025 mM AlCl_3_	−10.4 ± 0.8	9.8 ± 1.8
BSA + 20 mM NaCl + 0.05 mM AlCl_3_	−6.2 ± 0.5	9 ± 4
BSA + 20 mM NaCl + 0.1 mM AlCl_3_	–	9 ± 4
BSA + 20 mM NaCl + 0.3 mM AlCl_3_	–	9.9 ± 2.7
BSA + 20 mM NaCl + 0.4 mM AlCl_3_	8.1 ± 1.0	11.3 ± 0.8*

*Statistically significant differences at p = 0.00004.

As can be seen from Table 1, with the increase in the concentration of AlCl_3_ and FeCl_3_ salts, the $\upzeta$-potential of BSA decreases in absolute value, and at 0.4 mM AlCl_3_ it changes sign. The measurement of the $\upzeta$-potential at 0.1 mM FeCl_3_ and higher is not possible due to the presence of colloidal particles larger than 100 nm in diameter (most likely formed by iron hydroxide). A decrease in the electrokinetic potential of protein particles is due to a decrease in pH and screening of their surface potential by electrolyte; according to^2^, pH change plays a major role.

Below the 0.1 mM Fe^3+^ concentrations (at least up to 0.05 mM), particles of non-aggregated proteins with a diameter of 9–10 nm predominate. The addition of Al^3+^ to concentrations of 0.4 mM leads to an increase in the size of protein particles approximately by 2 nm; however, in solutions with an Al^3+^ concentration of 0.1–0.3 mM, signs of BSA aggregation and sedimentation were observed. In this range of Al^3+^ concentrations, the potential of BSA particles is close to zero, which promotes the aggregation of protein molecules. These particles can be formed by denatured protein aggregates^41^, which are also condensation centers for part of the native protein in the dispersion.

Thus, the decrease in L_count_ and L_specific_ may be attributed to an increase in the amount of protein that does not take part in the formation of these textures due to protein denaturation and condensation in the process of self-organization^42^. For Fe^3+^, it is due to protein condensation (aggregation) on colloidal particles of iron hydroxides both in BSA solutions and during their evaporation. For Al^3+^, it correlates with the onset of precipitation in BSA solutions when Al^3+^ concentration raises above 0.1 mM, which may be associated with recurrent condensation in solutions due to a considerable decrease in the absolute value of the protein surface potential^10^. The aggregation of the protein also can explain the decrease in the BSA hydration. Thus, we can conclude that the dominant factor in the change in L_count_ and L_specific_ is the change in the concentration of Al^3+^ and Fe^3+^ ions.

### Fluorescence spectra measurements of BSA solutions with Al^3+^ and Fe^3+^, and films with Fe^3+^

**Figure 4:**
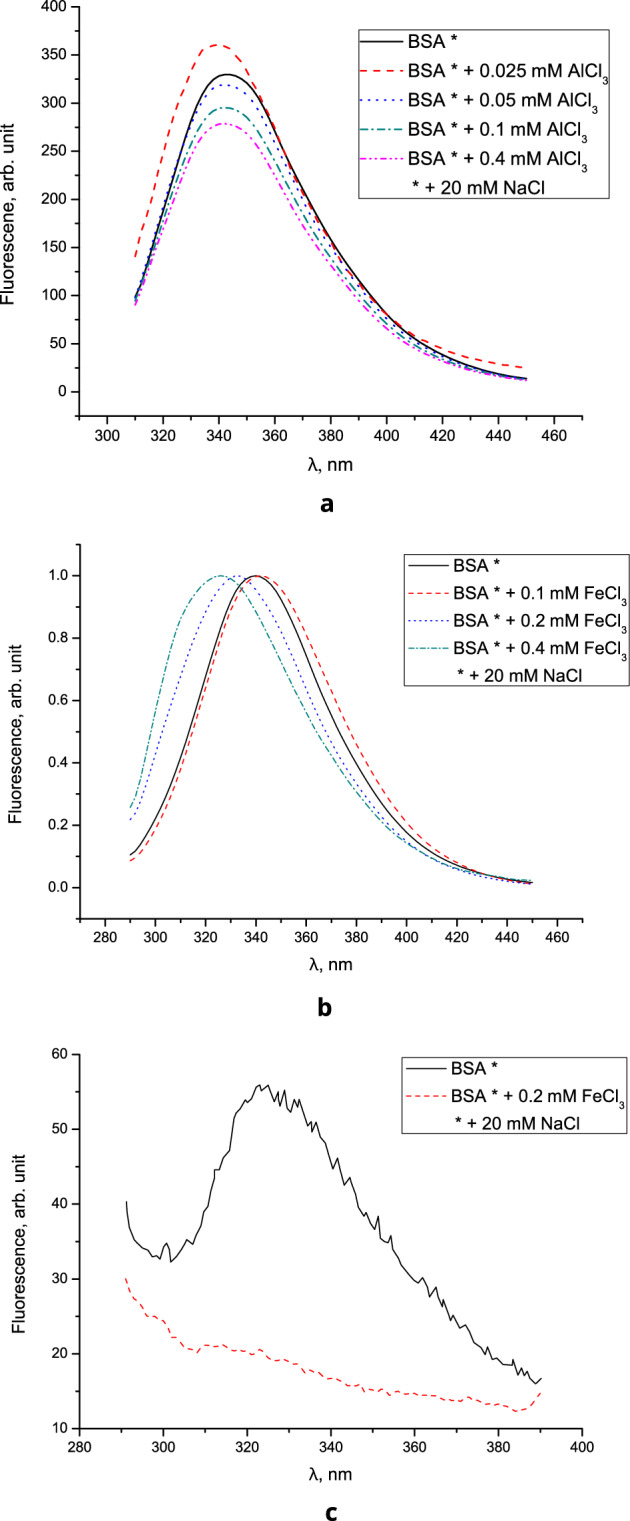
(**a**) Fluorescence spectra of BSA solutions with AlCl_3_, (**b**) normalized fluorescence spectra of BSA control solution and BSA solutions containing Fe^3+^ ions, (**c**) fluorescence spectra of films from solutions.

The fluorescence spectra of BSA solutions with AlCl_3_ are shown in Fig. 4a. It can be seen from the spectra that, with the addition of AlCl_3_ at a concentration of 0.025 mM, an increase in the fluorescence intensity is observed, and then, with an increase in the AlCl_3_ concentration to 0.05 mM, the fluorescence intensity decreases.

It is known that up to three Al^3+^ ions^8^ in two coordination sites^9^ bind to BSA, while chemical bonds can be formed both with nitrogen and oxygen atoms on the peptide backbone and with charged side chains of amino acids on the protein surface^2,10^.

It is known that two tryptophan residues Trp-134 and Trp-212, located near the surface of the IB domain and in the hydrophobic interior of the IIA domain, respectively, make a substantial contribution to protein fluorescence^43^. The fluorescence intensity can decrease (fluorescence quenching) or increase as a result of the interaction of BSA with ions. Thus, a change in the fluorescence intensity indicates a change in the microenvironment around the chromophore molecule. The decrease in the fluorescence intensity can apparently be explained by the quenching of the Trp-134 fluorescence in the presence of Al^3+^.

A considerable decrease in the fluorescence intensity at a concentration of aluminum ions C_Al_ > 0.05 mM can be explained by the precipitation of BSA. An increase in turbidity, as well as reenterability, is characteristic of BSA solutions containing trivalent ions^44,45^.

The fluorescence spectra of BSA solutions obtained from the control solution and solutions containing Fe^3+^ ions at concentrations of 0.1–0.4 mM are shown in Fig. 4b. As can be seen, the hypsochromic shift is 350 cm^−1^ (from 342 nm maximum for BSA without Fe^3+^ to 332 and 326 nm for BSA solution containing 0.2 and 0.4 mM Fe^3+^, respectively). According to^46–48^, the fluorescence maximum at a wavelength of 340–342 nm corresponds to surface tryptophan residues in the protein in a hydrophilic microenvironment. Thus, a change in the position of the maximum indicates a change in the state of the hydrophilic environment of the protein globule.

The fluorescence spectra of the films obtained from the control solution and the solution containing Fe^3+^ ions are shown in Fig. 4c. The maximum of the fluorescence band of the films is at 327 nm. As follows from Fig. 4c, the fluorescence intensity of the film obtained from the control solution is much higher than that of the film obtained from the solution containing Fe^3+^ ions. The fluorescence maximum of the BSA film in the presence of Fe^3+^ is hypsochromically shifted by 1990 cm^−1^. A decrease in the fluorescence intensity and a shift in the maximum of the BSA emission band in the presence of Fe^3+^ ions can be explained both by a change in the conformation of protein molecules and by partial reabsorption of the fluorescence of tryptophan amino acid residues in BSA molecules by Fe^3+^ ions. Thus, the analysis of the fluorescence spectra does not allow us to make an unambiguous conclusion about the effect of iron ions on the structure of BSA globules.

### Measurements of absorption spectra of BSA solutions with Al^3+^ and Fe^3+^

**Figure 5:**
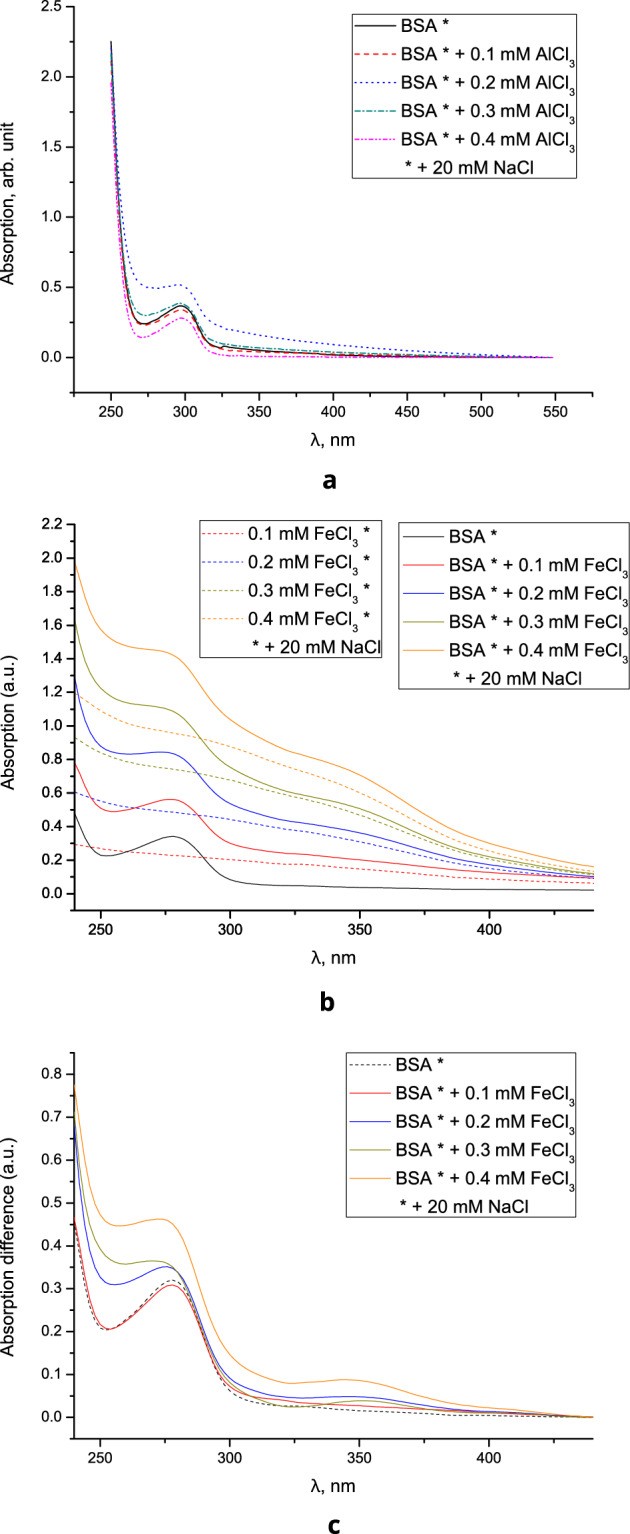
(**a**) UV absorption spectra of BSA + Al^3+^ solutions, (**b**) UV absorption spectra of Fe^3+^ and BSA + Fe^3+^ solutions, (**c**) difference between the BSA + Fe^3+^ spectra and the corresponding Fe^3+^ spectra.

UV absorption spectra of BSA solutions with AlCl_3_ are shown in Fig. 5a. The change in the absorption intensity at different concentrations of iron and aluminum salts can be explained by the effects associated with the metastable state of the protein solution—due to the influence of pH change caused by the hydrolysis of metal salts and the inversion of the protein charge^2^, and also, in the case of solutions with aluminum ions, due to interaction with main and side chains with possible denaturation^41^.

The UV absorption spectra of the Fe^3+^ and BSA + Fe^3+^ solutions, as well as the difference between the BSA + Fe^3+^ spectra and the corresponding Fe^3+^ spectra, are shown in Fig. 5b, c. An analysis of the spectral curves has shown that, in the presence of iron ions, BSA solutions absorb more intensively in the short-wavelength region. As can be seen from Table 2, the absorption maximum of solutions containing Fe^3+^ at a concentration of 0.4 mM is hypsochromically shifted relative to the absorption maximum of the BSA control solution. Through a twofold differentiation of the spectral curves for these solutions, the refined values of the absorption band maxima were obtained, which helped to determine that the hypsochromic shift is 525 cm^−1^ (from 278 nm maximum for BSA without Fe^3+^ to 274 nm for BSA solution containing Fe^3+^ at maximum concentration).

**Table 2:** Parameters of absorption and fluorescence spectra of BSA samples containing Fe^3+^ ions in various concentrations (mean ± standard deviation).

Fe^3+^ concentration, mM	Maximum absorption intensity, relative units	Wavelength of the position of the maximum of the absorption spectrum, nm	Wavelength of the position of the maximum of the fluorescence spectrum, nm
0	0.32 ± 0.03	278 ± 2	342 ± 2
0.1	0.33 ± 0.02	278 ± 2	342 ± 3
0.2	0.35 ± 0.03	275 ± 2	332 ± 2
0.4	0.48 ± 0.02	274 ± 2	326 ± 2

This spectral effect indicates that the introduction of ferric ions into the corresponding binding site (the concentration of Fe^3+^ substantially exceeds the concentration of protein molecules) leads to a change in the tryptophan environment in the BSA globule and, possibly, to a partial change in its helical and tertiary structure. This conclusion is consistent with the conclusions given in^7^.

## Summary

Based on our previous studies^36,49,50^, the decrease in the number of Z-structures on films obtained from BSA solutions can be due to the following reasons. Firstly, this may be due to a change in the charge and size of BSA particles^50^. Secondly, due to a change in conformation or a violation of the structure of BSA^36,50^. Thirdly, due to a change in the concentration of sodium chloride, the presence of other salts in the solution^36^. These factors, in turn, affect the hydration of the solution components and the structural state of free water in solution, which presumably also affects the formation of Z-structures^36^.

Let us consider how the presence of AlCl_3_ and FeCl_3_ salts affects the above causes.

When adding 0.025 and 0.05 mM AlCl_3_ to BSA + NaCl solutions, the $\upzeta$-potential of BSA decreased in absolute value by 5 mV and 9.6 mV relative to the control, respectively, and changed sign at 0.4 mM AlCl_3_ (Table 1). The addition of AlCl_3_ at concentrations of 0.025, 0.05, and 0.4 mM leads to an increase in the size of protein particles approximately by 2 nm; however, in solutions with 0.1–0.3 mM AlCl_3_, signs of BSA aggregation and sedimentation were observed (in this AlCl_3_ concentration range the $\upzeta$-potential is close to zero, which contributes to the aggregation of protein molecules; however, it can be assumed that the degree of protein aggregation at 0.1–0.3 mM AlCl_3_ is not great enough to violate the crystallization conditions). The addition of 0.025 and 0.05 mM AlCl_3_ also leads to an increase in the intensity of BSA fluorescence by 10–30% (Fig. 4a), which may be due AlCl_3_ hydrolysis, as well as due to interaction with the protein backbone and amino acid side chains^10,41^.

Amplification of these structural changes with an increase in the concentration of AlCl_3_ to 0.4 mM and a decrease in pH can cause a decrease in the number of segments of zigzag patterns starting from a concentration of 0.4 mM AlCl_3_.

The decrease in L_count_ and L_specific_ correlates with the onset of BSA precipitation in solutions at pH < 3.7 (AlCl_3_) and pH < 3 (FeCl_3_), which may be due to the reentrancy of solutions due to a considerable decrease in the absolute value of the protein surface potential^10^. Thus, the dominant factor in the change in L_count_ and L_specific_ is the change in the concentration of Al^3+^ and Fe^3+^ ions.

It can be noted that an increase in the standard deviation (scatter dispersion) in the absorption and fluorescence spectra can also be associated with the reentrancy (instability) of BSA solutions with trivalent metal ions^2,44^.

A DLS study^51^ showed that, even in the absence of proteins, FeCl_3_ solutions with a concentration of 0.1 mM or higher contain positively charged colloidal particles with a diameter of more than 100 nm (most likely formed by iron hydroxide). This can lead to further errors in the interpretation of DLS data, therefore, the study of protein solutions with FeCl_3_ was carried out only for FeCl_3_ concentrations of 0.025 and 0.05 mm. According to DLS measurements, in the studied solutions at Fe^3+^ concentrations up to 0.05 mM, particles of non-aggregated proteins with a diameter of 9–10 nm predominate. The $\upzeta$-potential of the particles decreases with increasing concentration of Fe^3+^ ($\upzeta$_0_ = −20 ± 3 mV, $\upzeta$_0.025_ = −14 ± 5 mV, $\upzeta$_0.05_ = −12 ± 5 mV), which is a manifestation of screening of the surface potential by the electrolyte solution, typical for colloidal systems. In addition, the pH value decreases by 0.5–2 units due to salt hydrolysis, which also affects the neutralization of the BSA charge.

The addition of FeCl_3_ leads to a slight hypochromic shift of the long-wavelength BSA absorption band from 278 nm (BSA solution without FeCl_3_) to 274 nm (BSA solution in the presence of 0.4 mM FeCl_3_)^51^ and to an increase in UV absorption intensity^52^ (Fig. 5c).

This can be caused either by protein unfolding (due to changes in hydration or pH) or by a pH-induced change in colloidal iron^53^ (resulting in an increase in background absorbance). The emission band in the BSA fluorescence spectrum also experiences a hypochromic shift from 342 nm (BSA solution without FeCl_3_) to 332 and 326 nm for BSA solutions containing 0.2 and 0.4 mM FeCl_3_, respectively^51^. The shift of the emission band can be associated both with structural changes in protein molecules and with partial reabsorption of the fluorescence of tryptophan amino acid residues in BSA molecules by Fe^3+^ ions. As a result, based on the UV absorption and fluorescence spectra, it is impossible to draw an unambiguous conclusion about the effect of Fe^3+^ on the BSA structure.

The decrease in the number of segments of zigzag patterns at concentrations ≥ 0.2 mM for FeCl_3_ and ≥ 0.4 mM for AlCl_3_ may be associated with structural changes in BSA that affect the dynamics of particle aggregation into structures on substrate surface when drying. This can lead to an increase in the polydispersity of BSA particles and a deterioration in crystallization^54–56^. But since iron hydroxide also forms colloidal particles, the polydispersity of particles in solution and the deterioration of crystallization are not necessarily associated only with protein aggregation.

A decrease in the degree of BSA hydration in solutions with NaCl + AlCl_3_ and NaCl + FeCl_3_ salts, as well as a more ordered structure of free water in them, can also play an important role in reducing the number of Z-structures in patterns. After evaporation of all free water in samples with AlCl_3_ and FeCl_3_ salts, there will probably be more competition for hydration water between the dissolved components. This, in turn, will determine the nature of electrostatic interactions in the system and the nature of the interaction between its components. Ultimately, these interactions manifest themselves at the macro level in the form of various types of textures that form on the surface of the dried films.

## Conclusions

It has been shown that Al^3+^ and Fe^3+^ metal ions change pH and make free water more structured in BSA solutions with NaCl, change the structural state and reduce the degree of BSA hydration, affect the specific length and the number of zigzag segments on the film surface.

When aluminum was added to BSA solutions with NaCl, pH decreased and the surface potential of the protein was compensated; however, the fluorescence intensity, as well as specific length and number of segments of zigzag segments at low aluminum concentrations changed nonmonotonically.

The change in the parameters of UV spectra in the presence of aluminum ions is mainly determined by the interaction of aluminum ions with BSA. The fluorescence spectra indicate a change in protein conformation in the presence of AlCl_3_; however, the fact that the number of zigzag segments does not decrease in this case, most likely, indicates that these aluminum concentrations do not lead to substantial inhomogeneity of the protein structure.

Comparison of changes in the number of zigzag segments on films with changes in the structural state of the protein in solution upon the addition of various iron concentrations has shown that, in this case, the number of zigzag segments changes, but not their geometrical parameters. At the same time, an increase in iron concentration leads to the predominance of other types of patterns.

With an increase in the concentration of iron, a decrease in pH occurs as a result of iron hydroxylation, in turn leading to the screening of the protein surface potential, which can contribute to the aggregation of protein particles. But since iron hydroxide itself forms colloidal particles, the polydispersity of the solution and the deterioration of crystallization are not necessarily associated with protein aggregation.

The results obtained can be useful for evaluating the effect of coagulants. It is also of interest to study changes in the parameters of patterns related to the influence of aluminum and iron ions on other proteins in conjunction with the state of tissues and organs of the body.

## Methods

### Z-pattern method

The method used in this work for assessing the effect of biologically active substances on biopolymers consists of drying a solution of a biopolymer with BAS and determining the number of Z-patterns from photographs of textures on a film. A decrease in this value determines the degree of influence of biologically active substances on the biopolymer in solution^31^.

Solutions of 0.5 mg/ml BSA (DiaM, USA) with 20 mM NaCl in distilled water were used, with the addition of aluminum (up to 0.8 mM) and iron chlorides (up to 0.4 mM).

The films were obtained by drying 0.5 ml of the solution in a 20 × 20 × 1 mm^3^ glass cell for 3 h (at a temperature of 40 ± 0.5 °C, 0.5 ± 0.1 atm. air pressure and 5 ± 3% relative humidity).

After drying, the cuvettes with films were removed from the drying chamber and placed in sealed glass boxes with silica gel for subsequent photography.

Micrographs of the films were taken at a 10× zoom, with focus adjusted for maximal image sharpness, using a webcam attached to a microscope (Meopta-Optika, Czech Republic); the images were taken at a resolution of 640 × 480 pixels and using image averaging to reduce the noise. Each film was photographed at 100 positions evenly distributed over the cuvette area in a 10 × 10 square grid manner.

**Figure 6:**
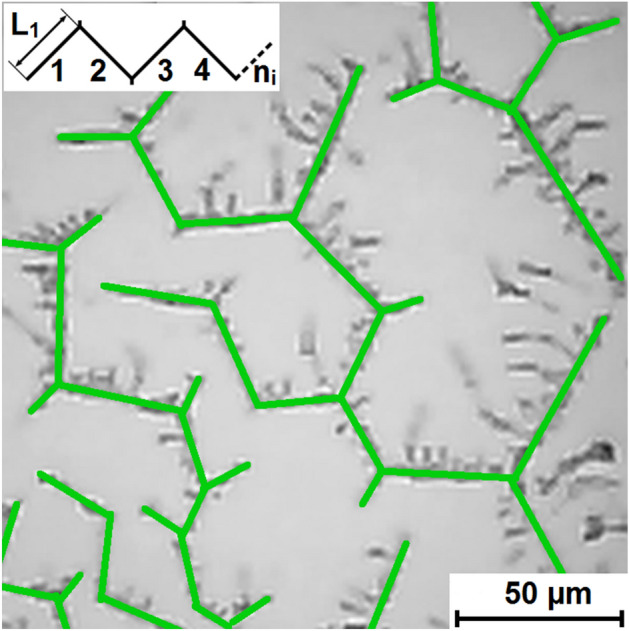
A film with zigzag textures obtained from a solution of 0.5 mg/ml BSA + 20 mM NaCl. Zigzag segments are marked in green. The length of an i-th segment is **L**_**i**_ and the number of segments is **n**.

Using a custom software, segments of Z-patterns were manually marked on each micrograph (Fig. 6). To characterize Z-patterns, parameters such as the length and number of segments were used.

Based on this data, the specific length (L_specific_)^36,49^ and the total number (L_count_)^49^ of segments were calculated for each film:

1 $${L}_{count}={\sum }_{p=1}^{N}{n}_{p},$$

2 $${L}_{sum(p)}={\sum }_{i=1}^{{n}_{p}}{L}_{i},$$

3 $${L}_{specific}=\frac{1}{{S}_{p}\cdot N}{\sum }_{p=1}^{N}{L}_{sum \,(p)},$$

where *N* is the number of micrographs, *n*_*p*_ is the number of segments in micrograph *p*, *L*_*i*_ is the length of segment *i*, *S*_*p*_ is the area of micrograph *p*.

The values obtained for 4**–**6 individual films were then averaged.

To estimate the statistical significance (at p < 0.05) of the correlations between the characteristics of zigzag patterns and the concentrations of AlCl_3_ and FeCl_3_, the p-values for the non-parametric Spearman's rank correlation were calculated via a two-sided permutation test (with sample size of n = 43 for AlCl_3_ and n = 52 for FeCl_3_).

### Microwave dielectrometry

The state of the water was studied by a method based on measurement of the dielectric permittivity of solutions at the microwave frequency using a cylindrical resonator with the H_01n_ oscillation type. The method is described in^37^ in detail.

The dielectric permittivity is a complex value: ${\varepsilon }^{*}={\varepsilon }^{{\prime}}-i{\varepsilon }^{\prime \prime}$, where the real part ${\varepsilon }{^{\prime}}$ is the ability of the material to be polarized by an external electric field, and the imaginary part ${\varepsilon }^{\prime \prime}$ is proportional to the energy absorbed from the field (dielectric losses). The frequency dependences of ${\varepsilon }{^{\prime}}$ and ${\varepsilon }^{\prime \prime}$ for pure water and protein solutions in the microwave region can be described by the equations^57,58^. The peak in ${\varepsilon }^{\prime \prime}$ occurs at the relaxation frequency ${f}_{d}$ (which is 14.5 GHz for pure water at 15.5 °C). The reciprocal of $2\uppi {f}_{d}$ is the relaxation time ${\uptau }_{1}$. The relaxation properties of water can be considered in the light of a wait-and-switch model of dielectric relaxation, in which the relaxation time is governed by the period for which a given ensemble of hydrogen bond partners within the hydrogen network has to wait until favorable conditions arise for the reorientation of a molecular permanent dipole^38^.

The measured dielectric losses consist of a dipolar component and the ionic losses due to the conductivity of ions of salts. At low frequencies, ionic conduction is the most prevalent mechanism. In this case ${\varepsilon }_{measured}^{ \prime \prime } ={\varepsilon }^{\prime \prime} +\frac{\sigma }{2\pi {\varepsilon }_{0}f}$, where $\sigma$ is the ionic conductivity of dissolved salts, $f$ is the frequency, ${\varepsilon }_{0}$ is the dielectric constant of the free space (${\varepsilon }_{0}=8.854\times {10}^{-12}{\mathrm{F}}^{-1}{\mathrm{m}}^{-1}$)^57,58^. In order to exclude the ionic losses, the corrections were made based on measurements of electrical conductivity $\sigma$ at a frequency of 1 kHz. In the following calculations, we have used the dielectric losses that consist only of the dipolar component (${\varepsilon }^{\prime \prime}$).

Water molecules in the protein solution may be classified into bound water that interacts with the protein and free (bulk) water^58^. In salt solutions there is bound water that interacts with ions, and free water. These types of water have different mobility, dispersion regions and dielectric permittivity^58^. We study the solutions in the region of relaxation of free water molecules ($\upgamma$-dispersion). The protein and bound water have low dielectric permittivity at the microwave frequency^58^. We use the frequency of 9.2 GHz in our experiment and can detect predominantly free water molecules. Conformational or structural changes in a macromolecule lead to the redistribution of free and bound water and, thus, to the change in the amount of free water molecules. Therefore, we can detect the changes in proteins using free water as a marker^37^. The static dielectric permittivity ${\varepsilon }_{s}$ of protein and salt solutions is less than that of pure water by the amount which is proportional to the amount of solute substance and bound water. This lowering can be used to obtain information concerning the amount of bound water (the protein hydration).

In our work, real ${\varepsilon }^{{\prime}}$ and imaginary $\varepsilon^{\prime \prime}$ parts of the complex permittivity of BSA solution (10 mg/ml) in pure water, salt solutions (0.4 M NaCl; 1 mM AlCl_3_; 1 mM FeCl_3_; 0.4 M NaCl and 1 mM AlCl_3_; 0.4 M NaCl and 1 mM FeCl_3_), and water-BSA-salt systems (10 mg/ml BSA and 0.4 M NaCl; 10 mg/ml BSA, 0.4 M NaCl, 1 mM AlCl_3_; 10 mg/mL BSA, 0.4 M NaCl, 1 mM FeCl_3_) were measured at 15.5 ± 0.1 °C. Corrections for the presence of inorganic ions were made based on measurements of electrical conductivity at a frequency of 1 kHz. The dielectric relaxation frequency *f*_*d*_ of water molecules in solutions and the static permittivity ${\varepsilon }_{s}$ were calculated from the obtained values of ${\varepsilon }^{{\prime}}$ and $\varepsilon ^{\prime \prime}$ using equations (4), (5) derived from the Debye equations^57,58^:

4 $${f}_{d}=\frac{f({\varepsilon }^{\prime}-{\varepsilon }_{\infty })}{\varepsilon^{\prime \prime}},$$

5 $${\varepsilon }_{s}=\varepsilon {^\prime}+\frac{\varepsilon ^{\prime \prime \,2 }}{{\varepsilon }^{\prime}-{\varepsilon }_{\infty }},$$

where *f* is the frequency of the microwave field, ${\varepsilon }_{\infty }$ = 5.6 is the permittivity of water in the infrared frequency range^59^.

### pH measurement

The pH measurements were carried out using a pH-150MI pH meter, equipped with a combined glass electrode ESK-10603/7 with a single-key reference electrode (Gomel, Belarus) The calibration of the electrode was performed with four standard buffer solutions (pH  1.68, 4.01, 6.86, and 9.18). All the measurements were carried out in the thermostat at 25 °C.

### Ultraviolet spectroscopy

A Hitachi U2310 spectrophotometer and a Hitachi 850 spectrofluorimeter (Hitachi, Ltd, Tokyo, Japan) were used to obtain absorption spectra (240**–**450 nm) and fluorescence spectra (290**–**460 nm, excitation at 280 nm). All spectra were recorded at 25 °C. To avoid the distortion of the spectral curves caused by the considerable light scattering from the film textures, a 290 nm cut-off filter was used to record the fluorescence spectra of the films.

### Light scattering techniques

The particle size was determined via dynamic light scattering, using the Zetasizer Nano ZS Malvern Instruments apparatus (Malvern, Great Britain), equipped with the He–Ne laser (633 nm), in disposable polystyrene cuvettes. The scattering angle was 173°, the attenuator value was 8–10, duration used as a rule was 70 s. The autocorrelation function was processed using the Zetasizer Software program, using a distribution fit. For each sample, 5–10 measurements with 12–40 runs were carried out at a temperature of 25 °C.

When necessary, samples were filtered through polytetrafluoroethylene filter with pore size of 0.2 $\upmu$m. The constancy of BSA concentration before and after filtration was proven using absorption spectra.

The $\upzeta$-potential was calculated from the electrophoretic mobility using the Smoluchowski approximation. The electrophoretic mobility was determined using electrophoretic light scattering at the Zetasizer Nano ZS Malvern Instruments apparatus in the folded capillary zeta cell DTS1070. As a rule, 5 measurements, 12–30 runs for each measurement, were used for calculations.

The diffusion barrier method was used to prevent electrocoagulation of the protein^60^.

To estimate the statistical significance (at p < 0.05) of the differences between the BSA diameters at different AlCl_3_ concentrations, two-sided Mann–Whitney test was used (with control of family-wise error rate via Holm-Bonferroni method, and of false discovery rate via Benjamini–Hochberg method). Sample sizes for the corresponding concentrations were: n_0.0_ = 30, n_0.025_ = 13, n_0.05_ = 11, n_0.1_ = 9, n_0.3_ = 7, n_0.4_ = 19.

## Supplementary Information

Supplementary Figure S1.

## Data Availability

The datasets used and/or analyzed during the current study are available from the corresponding author on reasonable request.
