# Supplementary material for: Influence of aluminum and iron chlorides on the parameters of zigzag patterns on films dried from BSA solutions
Source: Sci Rep. 2023 Jun 9;13:9426. doi: 10.1038/s41598-023-36515-4 (PMC10256847; doi:10.1038/s41598-023-36515-4)
Supplement: Supplementary file 1 — Supplementary Figure S1. [file 41598_2023_36515_MOESM1_ESM.pdf]

Influence of aluminum and iron chlorides on the parameters of zigzag patterns on films dried from BSA solutions

Dmitriy Glibitskiy[1,*], Olga Gorobchenko[2], Oleg Nikolov[2], Tatyana Cheipesh[2], Tatyana Dzhimieva[2], Inna Zaitseva[2,3], Alexander Roshal[4], Mihail Semenov[1], Gennadiy Glibitskiy[1]

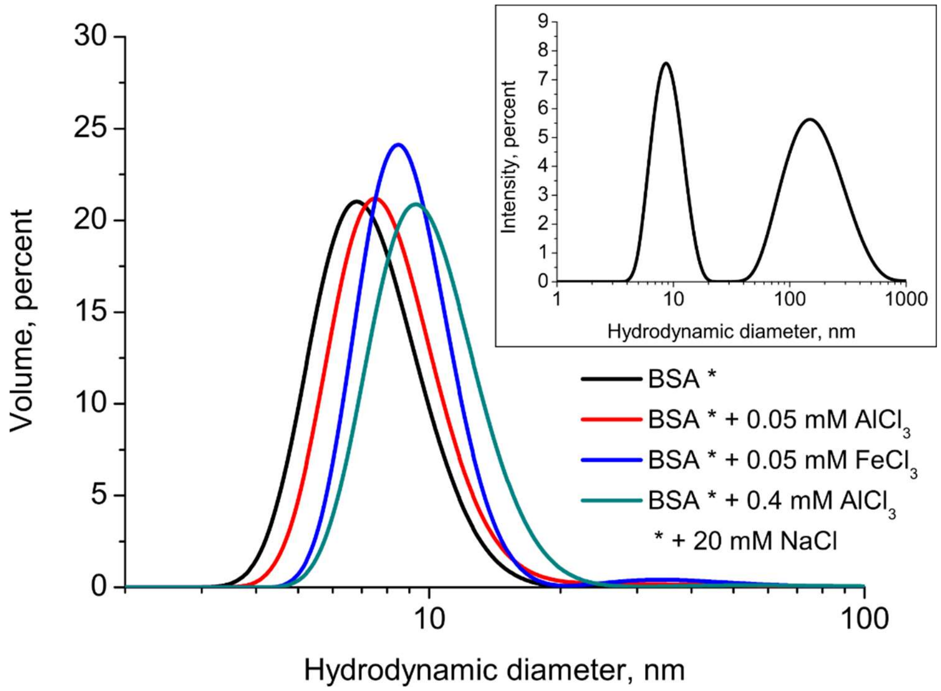

Supplementary Figure S1. Size distribution by volume and intensity (insert) of control BSA + 20 mM NaCl solutions and in the presence of $AlCl_3$ and $FeCl_3$.
